# Light intensity regulates phototaxis, foraging and righting behaviors of the sea urchin *Strongylocentrotus intermedius*

**DOI:** 10.7717/peerj.8001

**Published:** 2019-11-08

**Authors:** Jiangnan Sun, Xiaomei Chi, Mingfang Yang, Jingyun Ding, Dongtao Shi, Yushi Yu, Yaqing Chang, Chong Zhao

**Affiliations:** Key Laboratory of Mariculture & Stock Enhancement in North China’s Sea, Ministry of Agriculture and Rural Affairs, Dalian Ocean University, Dalian, China

**Keywords:** *Strongylocentrotus intermedius*, Light intensity, Phototaxis, Foraging, Righting behavior, Reseeding

## Abstract

Small sea urchins *Strongylocentrotus intermedius* (1–2 cm of test diameter) are exposed to different environments of light intensities after being reseeded to the sea bottom. With little information available about the behavioral responses of *S. intermedius* to different light intensities in the environment, we carried out an investigation on how *S. intermedius* is affected by three light intensity environments in terms of phototaxis, foraging and righting behaviors. They were no light (zero lx), low light intensity (24–209 lx) and high light intensity (252–2,280 lx). Light intensity had obvious different effects on phototaxis. In low light intensity, sea urchins moved more and spent significantly more time at the higher intensity (69–209 lx) (*P* = 0.046). *S. intermedius* in high light intensity, in contrast, spent significantly more time at lower intensity (252–690 lx) (*P* = 0.005). Unexpectedly, no significant difference of movement (average velocity and total distance covered) was found among the three light intensities (*P* > 0.05). Foraging behavior of *S. intermedius* was significantly different among the light intensities. In the no light environment, only three of ten *S. intermedius* found food within 7 min. In low light intensity, nine of 10 sea urchins showed successful foraging behavior to the food placed at 209 lx, which was significantly higher than the ratio of the number (two of 10) when food was placed at 24 lx (*P* = 0.005). In the high light intensity, in contrast, significantly less sea urchins (three of 10) found food placed at the higher light intensity (2,280 lx) compared with the lower light intensity (252 lx) (10/10, *P* = 0.003). Furthermore, *S. intermedius* showed significantly longer righting response time in the high light intensity compared with both no light (*P* = 0.001) and low light intensity (*P* = 0.031). No significant difference was found in righting behavior between no light and low light intensity (*P* = 0.892). The present study indicates that light intensity significantly affects phototaxis, foraging and righting behaviors of *S. intermedius* and that ~200 lx might be the appropriate light intensity for reseeding small *S. intermedius*.

## Introduction

The sea urchin *Strongylocentrotus intermedius* is a commercially important marine invertebrate in subtidal and shallow waters (Agatsuma, 2013). Increasing market demand has increased interest in the development of sea urchin reseeding. The annual production of sea urchins was around 10,000 tons in China 2017 (Zhang, 2018). Small *S. intermedius* (1–2 cm of test diameter) are reseeded to the sea bottom in both China (Lawrence, Zhao & Chang, 2019) and Japan (Agatsuma, 2013), where different light intensities occur from shallow water (0–20 lx) to intertidal zones (~2,200 lx). Unfortunately, little is known about the appropriate light intensities that affect the productivity of *S. intermedius*.

Phototaxis, foraging and righting are fitness-related behaviors, which can provide valuable information on appropriate light intensities for the reseeding of small *S. intermedius*. Phototaxis refers to the movement of organisms in response to light (Bendix, 1960). Light detection is essential for finding food, shelter and avoidance of predators of marine invertebrates (Kirwan et al., 2018), subsequently influencing their distribution (Tilman & Kareiva, 1997). Negative phototaxis has been well documented in sea urchins when they are exposed to high light intensity (Holmes, 1912; Yoshida, 1957; Ullrich-Lüter et al., 2011). Positive phototaxis was also found in sea urchins *Strongylocentrotus* (as *Allocentrotus*) *fragilis* and *Lytechinus variegatus*, although the light intensities were not recorded (Sharp & Gray, 1962; Salazar, 1970), respectively. However, it remains unknown of whether *S. intermedius* show positive or negative phototaxis in different light intensities and whether an appropriate light intensity for small *S. intermedius* exists. Efficient foraging is essential for the survival of the reseeded sea urchins, directly determining their productivity in the field (Agatsuma et al., 2019). 5.81 lx and 278 lx were two inflection points of daily activity rhythm of the sea cucumber *Apostichopus japonicas*. Relatively high light intensities (>278 lx) reduced the number of sea cucumbers that continued feeding (Dong et al., 2010). We hypothesized that light intensity significantly affects the foraging behavior of small *S. intermedius*. Righting, refers to the behavior of an inverted individual to resume the posture with aboral side up (Hyman, 1955), is essential for the marine invertebrates to escape from predation and from the effects of turbulence (Brothers & McClintock, 2015) and consequently important for reseeded sea urchins. Sea urchins showed significantly reduced righting behavior in environments of elevated temperature (Brothers & McClintock, 2015), chemical pollutants (Böttger, McClintock & Klinger, 2001), reduced salinity (Lawrence, 1975) and high *p*CO_2_ (Challener & McClintock, 2013). However, the effect of light intensity on righting behavior remains totally unknown, hampering our understanding of the potential survival risks of reseeded sea urchins.

The present study aims to investigate the effects of different light intensity environments on phototaxis, foraging and righting behaviors of small *S. intermedius*. We asked (1) whether light intensity significantly regulates positive and negative phototaxis of small *S. intermedius*; (2) whether foraging behavior of small *S. intermedius* significantly responds to light intensity in different light intensities; (3) whether light intensity significantly affects righting behavior of small *S. intermedius*; (4) What is the light intensity appropriate for small *S. intermedius* reseeding.

## Methods

### Sea urchins

Two hundred small *S. intermedius* were transported from Dalian Haibao Fishery Company (121°22′E 38°77′N, light intensity: 0–300 lx) to the Key Laboratory of Mariculture & Stock Enhancement in the North China’s Sea, Ministry of Agriculture and Rural Affairs, Dalian Ocean University (121°37′E 38°87′N). The sea urchins were 10.3 ± 1.3 mm of test diameter, 5.6 ± 0.7 mm of test height and 0.6 ± 0.2 g of body weight. There was no significant difference of test diameter, test height and body weight among sea urchins for all experiments. Sea urchins were maintained using a seawater temperature control system (Huixin Co., Dalian, China) in the laboratory with natural light ranging from zero to 1,500 lx and a constant water temperature at 15 ± 0.5 °C, fed fresh macroalgae *Ulva lactuca* for 2 weeks before the beginning of the experiments.

### Experimental design

**Figure 1:**
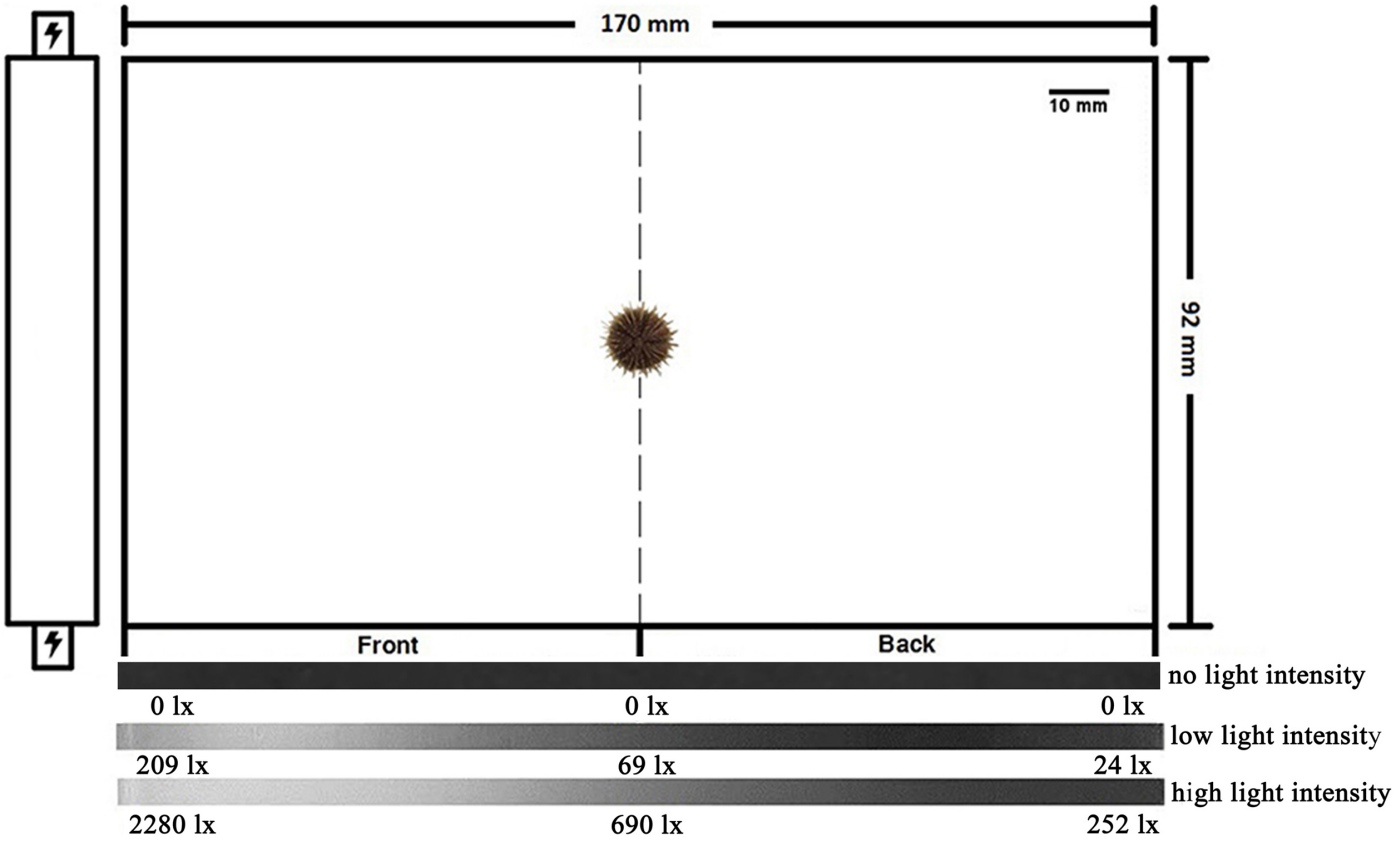
Diagram of the experiment aquarium. A lamp was placed in front of the aquarium. The aquarium was equally divided into two parts: front and back. The bar at the bottom of the diagram was taken from the experimental video recordings.

According to the method of Sharp & Gray (1962) with some revisions, the experiments were done in an acrylic aquarium (length × width × height: 170 × 92 × 50 mm) with a rod LED lamp (a cold light source with white light) on one side of the tank in a dark room (Fig. 1). The tank was equally divided into two parts: front and back (Fig. 1).

Light intensity varies in shallow water (0–20 lx) and intertidal water (~2,200 lx). Our preliminary experiment indicated that *S. intermedius* showed positive phototaxis at ~200 lx. Thus, three light intensities were set as no light (zero lx), low light intensity (24–209 lx) and high light intensity (252–2,280 lx). The light intensities in the middle of the aquarium were 69 lx and 690 lx at low light and high light intensities, respectively (Fig. 1). All behaviors were individually measured using different sea urchins in the aquarium at 15 °C. We changed the seawater and washed the aquarium for each trial to avoid potential non-experimental influences.

### Phototaxis

Sea urchins were placed individually in the center of the aquarium. We recorded movement by the sea urchin for 7 min using a camera (Legria HF20; Canon, Tokyo, Japan) (*N* = 5). Positive phototaxis refers to the movement of the sea urchin toward the light, while negative phototaxis is the opposite. We used the residence time (length of time in the front and back of the aquarium) to represent positive and/or negative phototaxis (Holmes, 1912). Distance and velocity were subsequently calculated using the track analysis in ImageJ (version 1.51 n).

### Foraging behavior

*Ulva lactuca* was cut into square pieces (10 × 10 mm) for each trial. In the low light intensity experiments, two pieces of *U. lactuca* were placed at 24 lx for one trial (*N* = 10) and at 209 lx for the other (*N* = 10). Similarly, two pieces of *U. lactuca* were placed at 252 lx for one trial (*N* = 10) and at 2,280 lx for the other (*N* = 10). Sea urchins were individually placed in the center of the aquarium at the beginning of each foraging trial. The number of sea urchins that reached the piece of *U. lactuca* within 7 min was recorded, using a camera (Legria HF20; Canon, Tokyo, Japan).

### Righting behavior

To prevent them from touching the wall of the tank, which affects righting (Shi et al., 2018), sea urchins were individually placed with their aboral side (the side opposite to the mouth) down in the center of the aquarium (20 mm depth). Righting response time, the time required for an individual to right itself with the aboral side up (Hyman, 1955), was individually recorded for each group (*N* = 10). If the sea urchin failed to right itself within 5 min, we set 300 s as the righting response time.

### Statistical analysis

Normal distribution and homogeneity of variance were analyzed using the Kolmogorov–Smirnov test and the Levene test, respectively. Average velocity and total distance were analyzed using one-way ANOVA. Residence time in phototaxis experiment and righting response time were analyzed using a Kruskal–Wallis one-way ANOVA, because of the abnormal distribution and/or heterogeneity of variance. Foraging behavior was analyzed using Fisher’s exact test. All statistical analysis was performed using SPSS 21.0 statistical software. A probability level of *P* < 0.05 was considered as significant.

## Results

### Phototaxis

**Figure 2:**
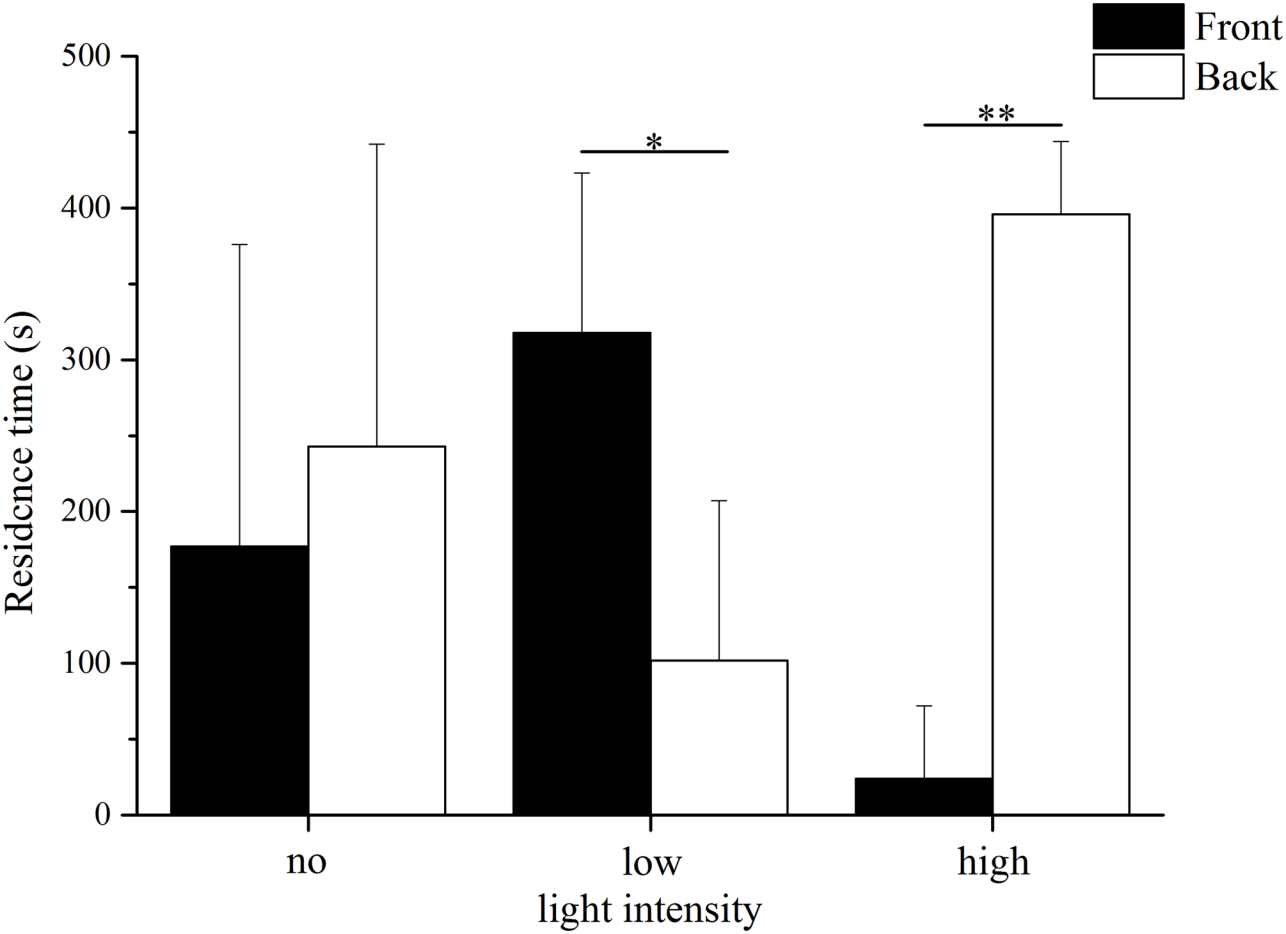
Residence time in front and back of the aquarium in no light, low light intensity and high light intensity (*N* = 5, mean ± SD). Significant differences are marked as * for *P* < 0.05, ** for *P* < 0.01.

The residence time in the different parts of the aquaria varied significantly between the low light intensity and high light intensity trials (Fig. 2). In low light intensity, sea urchins spent significantly more time in the front area (69–209 lx) than in the back area (24–69 lx) (front: 318.00 ± 105.15 s, back: 102.00 ± 105.15 s, Kruskal–Wallis *H* = 3.987, *P* = 0.046). In contrast, the residence time in the high light intensity trial was significantly longer in the back (252–690 lx) than in the front (690–2,280 lx) (front: 24 ± 48 s, back: 396 ± 48 s, Kruskal–Wallis *H* = 7.759, *P* = 0.005). No significant difference was found the residence time between the front and the back in darkness (front: 177.00 ± 199.09 s, back: 243.00 ± 199.09 s, Kruskal–Wallis *H* = 0.012, *P* = 0.911).

**Figure 3:**
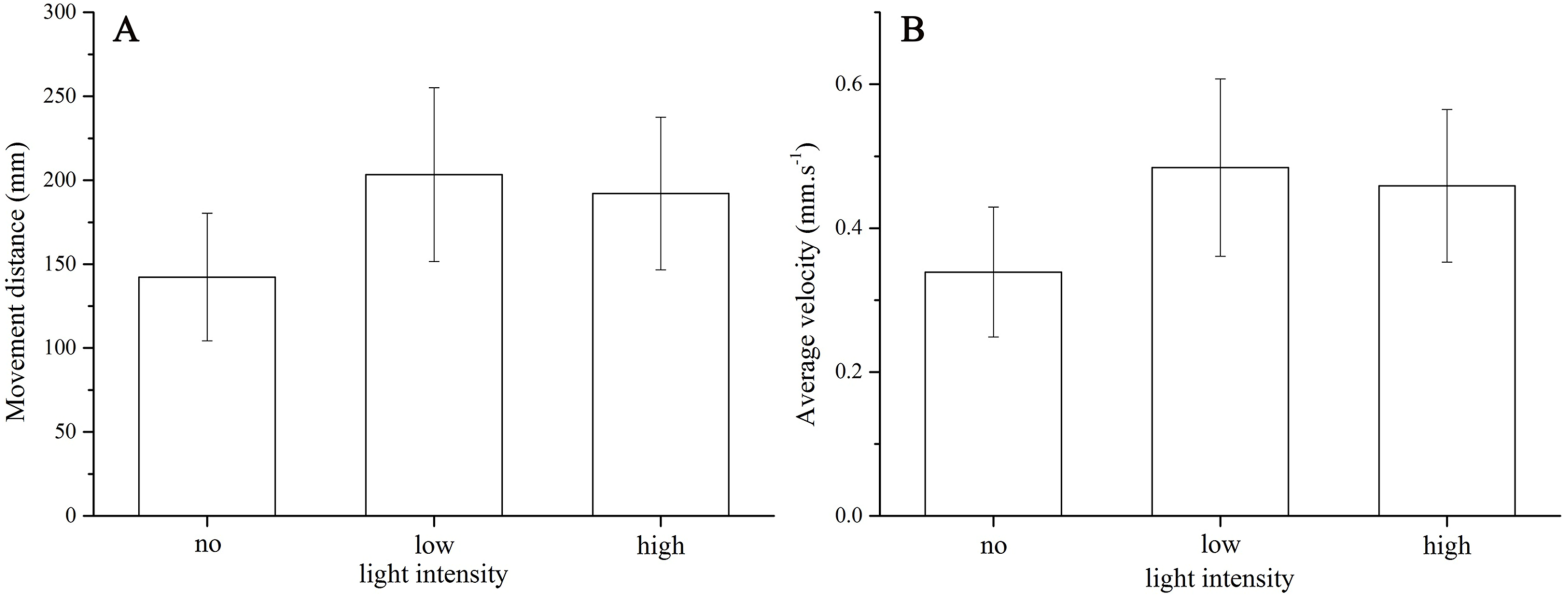
Movement distance (A) and average velocity (B) of *Strongylocentrotus intermedius* in no light, low light intensity and high light intensity.

*Strongylocentrotus intermedius* showed no significant difference of average velocity (no light: 0.34 ± 0.10 mm/s, low light intensity: 0.48 ± 0.14 mm/s, high light intensity: 0.46 ± 0.12 mm/s, *df* = 2, *F* = 2.060, *P* = 0.170) and moving distance (no light: 142.36 ± 42.49 mm, low light intensity: 203.38 ± 57.83 mm, high light intensity: 192.19 ± 50.80 mm, *df* = 2, *F* = 2.048, *P* = 0.172) among the three light intensities (Fig. 3).

### Foraging behavior

**Figure 4:**
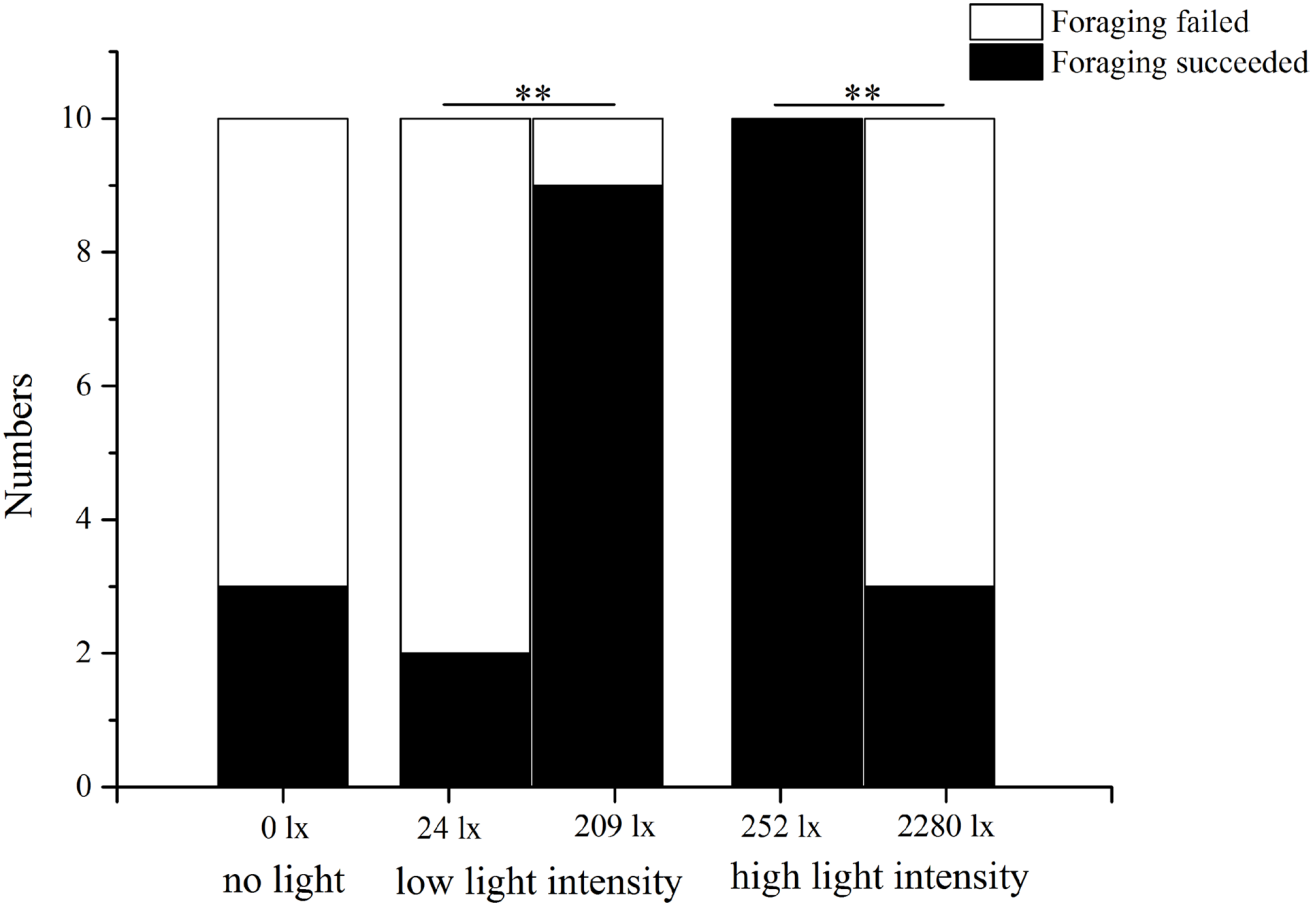
Number of foraging *Strongylocentrotus intermedius* in no light, low light intensity and high light intensity (*N* = 10, mean ± SD). Significant differences are marked ** for *P* < 0.01.

In the no light trial, only three of 10 *S. intermedius* found *U. lactuca* within 7 min. In low light intensity (209 lx), nine of 10 sea urchins found *U. lactuca*, which was significantly more than the number (two of 10) when food was placed at 24 lx (*P* = 0.005). In the high light intensity trial, in contrast, significantly less sea urchins (three of 10) found *U. lactuca* at the high light intensity (2,280 lx) than at the low light intensity (252 lx) (10/10, *P* = 0.003, Fig. 4).

### Righting response time

**Figure 5:**
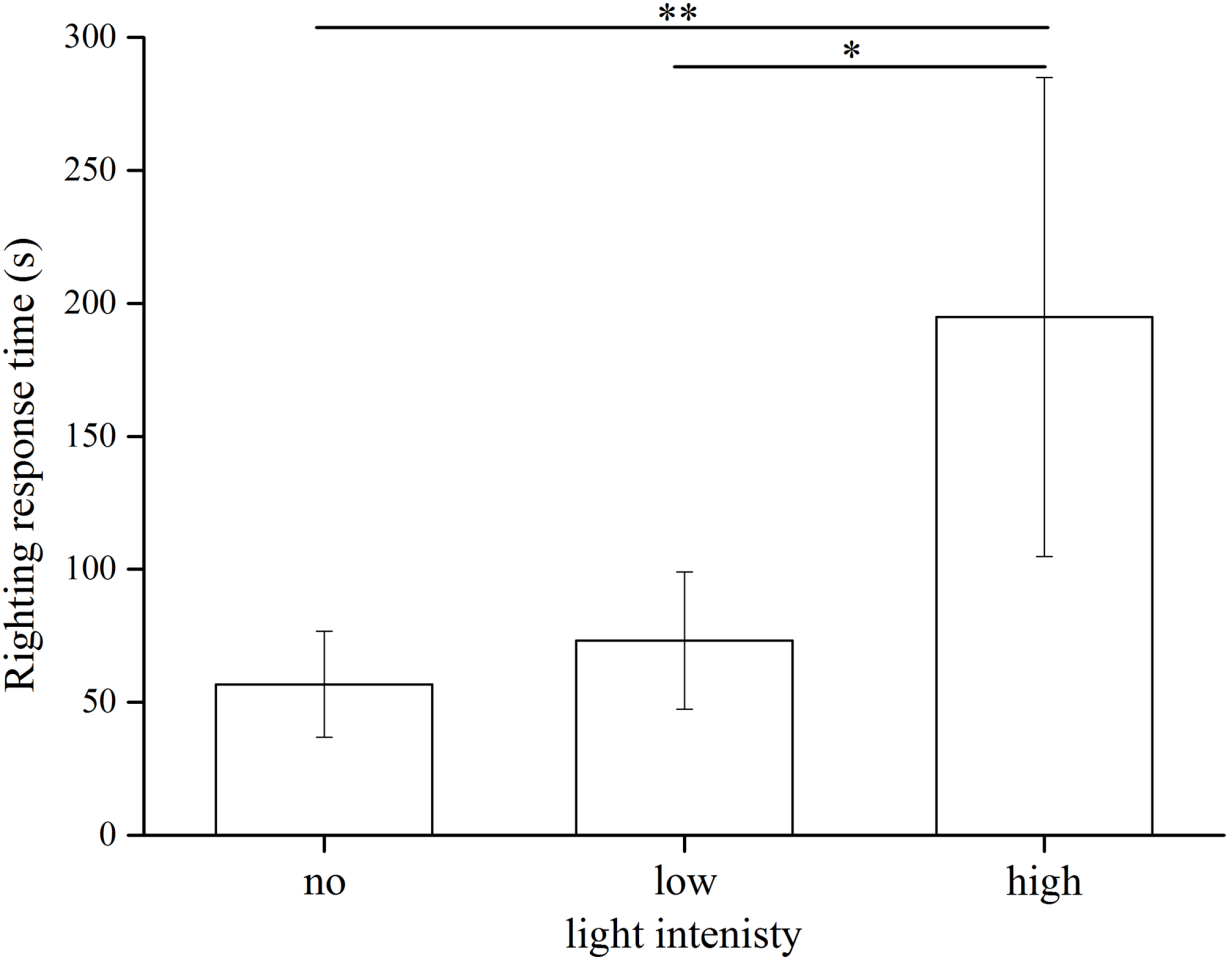
Righting time of *Strongylocentrotus intermedius* in no light, low light intensity and high light intensity (*N* = 10, mean ± SD). Significant differences are marked * for *P* < 0.05, ** for *P* < 0.01.

The statistical results showed significant differences among the three light intensities (Kruskal–Wallis *H* = 13.799, *df* = 2, *P* = 0.001). *S. intermedius* exposed to the high light intensity showed significantly longer righting time (194.94 ± 90.05 s) than those under the low light intensity (73.23 ± 25.79 s) (Kruskal–Wallis *H* = 10.100, *P* = 0.031) and no light (56.80 ± 19.85 s) (Kruskal–Wallis *H* = 14.200, *P* = 0.001) (Fig. 5). No significant difference was found between the no light and low light intensity (Kruskal–Wallis *H* = 4.100, *P* = 0.892).

## Discussion

Little is known of the effect of light intensity, which obviously differs in intertidal and shallow waters (McFarland, 1986), on sea urchins. This information of behavioral responses to light intensity sheds new light on the reseeding of sea urchins. For the first time, we show how light intensity regulates phototaxis, foraging and righting behaviors of sea urchins.

High light intensity induced negative phototaxis has been well documented in both field and laboratory (Holmes, 1912; Yoshida, 1957; Ullrich-Lüter et al., 2011). In this study, we consistently found an obvious negative phototaxis in small *S. intermedius* exposed to the high light intensity (252–2,280 lx). The result is consistent with the significantly increasing covering behavior at the high light intensity (Kehas, Theoharides & Gilbert, 2005), indicating the protective behavioral responses to the high light intensity. Since there is a trade-off between sheltering and foraging behaviors (Zhao et al., 2013), we hypothesized a subsequent impact of the high light intensity on the foraging behavior of *S. intermedius*. In this study, all sea urchins (10/10) foraged successfully at 252 lx. This can be explained by both food attraction and negative phototaxis. The behavior in response to the presence of food at different light intensities is in accordance with the finding of Fuji (1967) that food consumption significantly deceased when *S. intermedius* were exposed to the high light intensity. The current results indicate the negative effect of high light intensity on foraging behavior of sea urchins, although it is mainly based on chemical detection (Sakata et al., 1989). Considering the importance of foraging behavior to sea urchins (Agatsuma, Nakata & Matsuyama, 2000; Miayamoto & Koshima, 2006), we suggest small sea urchin not be reseeded in shallow water, where sea urchins would be exposed to high light intensity (e.g., 2,280 lx).

Here, a specific light intensity (209 lx) remarkably induced positive phototaxis, although it has been qualitatively described in field and laboratory studies (Sharp & Gray, 1962; Salazar, 1970). Light sensitivity allows sea urchins to detect different objects without eyes (Al-Wahaibi & Claereboudt, 2017). Further, this extraocular vision could help sea urchins find shelter and food (Kirwan et al., 2018). In the foraging experiment, most sea urchins found food at 209 lx. This indicates that an appropriate light intensity at the sea bottom, such as 209 lx, can help sea urchins forage. These results agree with the findings that the light intensity regulated foraging behavior in other species (e.g., the primate *Callithrix geoffroyi*) (Caine, Osorio & Mundy, 2010), although the molecular basis remains largely unknown.

In this experiment, high light intensity significantly prolonged the righting response time of *S. intermedius*. Since tube feet around the aboral are more photosensitive (Lesser et al., 2011), the reduced righting behavior at the high light intensity can be explained by the decreasing ability of tube feet to adhere to the substrate, inhibiting the righting behavior. This is consistent with the finding of decreased righting behavior at high temperature (Percy, 1972, 1973). Because righting behavior is important for sea urchins to escape from predators or physical turbulence (Brothers & McClintock, 2015), it is appropriate to release small *S. intermedius* in the low light intensity environments (e.g., 24–209 lx), rather than in the high light intensity environments (e.g., 252–2,280 lx). Interestingly, light intensity did not significantly affect velocity of movement and movement distance of *S. intermedius*. This indicates that high light intensity had less effect on the tube feet of oral surface than that of aboral side. Studies have shown that sea urchins’ velocity were not affected by environmental pressures within a given range, such as water temperature and current velocity (Dumont, Himmelman & Robinson, 2007). These results suggest that sea urchins are capable of effective movement in complex environments. This indicates that small urchins released into high light intensity environments have the locomotion capability to the low light intensity.

## Conclusion

High light intensity (252–2,280 lx) significantly induced negative phototaxis and hampered righting and foraging behaviors. Low light intensity (24–209 lx), to the contrary, significantly induced positive phototaxis and benefited righting and foraging behaviors. Small *S. intermedius* have the locomotion capability to the suitable light intensity environments. According to this study, we propose that ~200 lx is probably the appropriate light intensity for *S. intermedius* reseeding and suggest that aqua-farmers reseed small *S. intermedius* to the light intensity of ~200 lx. However, it should be noted that the present study is a laboratory-based investigation using artificial light. Thus, the field experiments during the enhancement of small *S. intermedius* are essential to further investigate the light intensity regulation of urchin behaviors and the importance of ~200 lx in sea urchin reseeding.

## Supplemental Information

10.7717/peerj.8001/supp-1 Supplemental Information 1 The size, speed, movement distance and residence time of sea urchins in the phototaxis experiment.

10.7717/peerj.8001/supp-2 Supplemental Information 2 The size and foraging time of sea urchins in the foraging experiment. TD, test diameter (mm); TH, test height (mm); BW, body weight (g).

10.7717/peerj.8001/supp-3 Supplemental Information 3 The size and righting response time of sea urchins in the righting experiment. TD, test diameter (mm); TH, test height (mm); BW, body weight (g).

## Additional Information and Declarations

### Data Availability

The following information was supplied regarding data availability: Raw data is available in the Supplemental Files.
